# Global Trends in Kidney Stone Awareness: A Time Series Analysis from 2004–2023

**DOI:** 10.3390/clinpract14030072

**Published:** 2024-05-20

**Authors:** Noppawit Aiumtrakul, Charat Thongprayoon, Supawadee Suppadungsuk, Pajaree Krisanapan, Preyarat Pinthusopon, Michael A. Mao, Chinnawat Arayangkool, Kristine B. Vo, Chalothorn Wannaphut, Jing Miao, Wisit Cheungpasitporn

**Affiliations:** 1 Department of Medicine, John A. Burns School of Medicine, University of Hawaii, Honolulu, HI 96813, USA; noppawit@hawaii.edu (N.A.); carayang@hawaii.edu (C.A.); kbvo@hawaii.edu (K.B.V.); cwanna@hawaii.edu (C.W.); 2 Division of Nephrology and Hypertension, Department of Medicine, Mayo Clinic, Rochester, MN 55905, USA; thongprayoon.charat@mayo.edu (C.T.); supawadee.sup@mahidol.ac.th (S.S.); pajareek@tu.ac.th (P.K.); miao.jing@mayo.edu (J.M.); 3 Chakri Naruebodindra Medical Institute, Faculty of Medicine, Ramathibodi Hospital, Mahidol University, Samut Prakan 10540, Thailand; 4 Department of Internal Medicine, Faculty of Medicine, Thammasat University, Pathum Thani 12120, Thailand; 5 Saint Joseph Convent School, Bangkok 10500, Thailand; 34484@sjc.ac.th; 6 Division of Nephrology and Hypertension, Department of Medicine, Mayo Clinic, Jacksonville, FL 32224, USA; mao.michael@mayo.edu

**Keywords:** kidney stone, nephrolithiasis, global trends, trends, time series, public awareness

## Abstract

Background: Despite the prevalence and incidence of kidney stones progressively increasing worldwide, public awareness of this condition remains unclear. Understanding trends of awareness can assist healthcare professionals and policymakers in planning and implementing targeted health interventions. This study investigated online search interest in “kidney stone” by analyzing Google Trends, focusing on stationarity of the trends and predicting future trends. Methods: We performed time series analysis on worldwide Google monthly search data from January 2004 to November 2023. The Augmented Dickey–Fuller (ADF) test was used to assess the stationarity of the data, with a *p*-value below 0.05 indicating stationarity. Time series forecasting was performed using the autoregressive integrated moving average to predict future trends. Results: The highest search interest for “kidney stone” (score 100) was in August 2022, while the lowest was in December 2007 (score 36). As of November 2023, search interest remained high, at 92. The ADF test was significant (*p* = 0.023), confirming data stationarity. The time series forecasting projected continued high public interest, likely reflecting ongoing concern and awareness. Notably, diverse regions such as Iran, the Philippines, Ecuador, the United States, and Nepal showed significant interest, suggesting widespread awareness of nephrolithiasis. Conclusion: This study highlighted that “kidney stone” is a consistently relevant health issue globally. The increase and stationarity of search trends, the forecasted sustained interest, and diverse regional interest emphasize the need for collaborative research and educational initiatives. This study’s analysis serves as a valuable tool for shaping future healthcare policies and research directions in addressing nephrolithiasis related health challenges.

## 1. Introduction

The worldwide prevalence and incidence of kidney stones is progressively increasing in individuals across diverse racial and age groups [1,2]. In the United States, the prevalence of nephrolithiasis rose from 3.2% in the 1970s to 8.8% in the early 2000s. This has been correlated with the increasing rates of metabolic diseases, such as obesity and type 2 diabetes mellitus [3,4,5]. The global prevalence of kidney stones also exhibits regional variations, with rates of approximately 9.3% in North America [6], 3.96% in South America [7], 4.7–6.8% in Europe [8,9,10], and 1.9–17.6% in Asia [11,12,13,14]. Studies [5,6,15,16] reporting prevalence and incidence of kidney stones often exhibit inconsistencies, which may be attributed to genetic factors [17], demographic variations, geographical distinctions, lifestyle differences [2], and especially an absence of a standardized kidney stone classification system [18].

While the exact pathophysiology of stone formation is unclear, the initial crucial step is the supersaturation of urine by stone constituents [19]. Various theories have been proposed as the pathophysiological mechanisms of urinary stone formation [20,21], but they all widely acknowledge that kidney stone formation is a multifactorial process influenced by demographics, geography, climate, diet, metabolism, and genetics. The physicochemical properties of drinking water may also play a role in kidney stone formation. Water with a specific composition, characterized by high magnesium and sulfate levels, has been found to lead to the formation of larger calcium oxalate crystals, while water with a different composition, featuring high bicarbonate, low magnesium, and low sulfate levels, resulted in smaller crystal precipitation [22]. In addition to water composition, specific factors significantly increasing the risk for nephrolithiasis include changing dietary patterns, such as high fructose consumption [23,24], and global warming [25,26]. The impact of global warming disproportionately increases nephrolithiasis risk in the population living in the “stone belt”, potentially increasing the risk from 40% in 2000 to 70% by 2095 [26]. Furthermore, individuals with osteoporosis were found to have a 1.33-fold higher risk for kidney stones compared to those without osteoporosis [27], highlighting the complex interplay between bone health and kidney stone formation.

As kidney stones become more prevalent, the deleterious impact on global health and quality of life increases. Patients with kidney stones suffer from recurrent severe renal colic pain and frequent medical visits that disrupt daily life, work, family time, and sleep [28,29]. Moreover, nephrolithiasis is linked to chronic kidney disease (CKD), with the mechanism primarily attributed to obstructive uropathy or pyelonephritis [30,31]. It can also increase the risk of developing end-stage renal disease [32], cardiovascular events [33], and stone-related mortality [34].

Medical treatments have benefited from research and technological advancements that have allowed earlier and more specific diagnosis of stone disease, individualized nonpharmacologic treatments, and innovative medication and surgical options. Patients with kidney stones generally require lifestyle modifications to prevent further stone formation, which include an increase in fluid intake, restriction of dietary sodium, limitation of animal protein, and ingestion of an appropriate amount of dietary calcium [3,35]. Interventions for stone removal have significantly changed, shifting from traditional open surgery in the early 1990s to contemporary minimally invasive procedures such as percutaneous nephrolithotomy and ureteroscopy. Recent innovations in this surgical field include advancements in laser technologies that provide enhanced precision, efficiency, and safety compared to prior technology [36]. 

Despite these advancements and efforts to curtail the prevalence and burden of kidney stone disease, a comprehensive study to assess the overall interest and concerns of the general population for kidney stones is lacking. Google Trends [37] is a web-based tool offered by Google that has been available since 2004. It analyzes the top search queries from Google searches across the regions of the world, and may provide actionable information to healthcare providers. Google Trends data could also provide a measure of the public interest and awareness of kidney stones over a period of time. Finally, analyzing Google Trends for kidney stones offers insights into the type and frequency of information patients seek. These data can allow healthcare providers and policymakers to deliver targeted education online, strategize clinical research and trials, and improve strategies to prevent and reduce consequences of kidney stones. 

This study aimed to analyze global public interest in kidney stones over nearly two decades from January 2004 to November 2023, utilizing data from Google Trends. By understanding these search patterns, the study results could help healthcare professionals and policymakers in developing targeted interventions.

## 2. Materials and Methods

This study involved the demonstration and analysis of online search trends of “kidney stone”. The Google Trends tool (https://trends.google.com/trends/ (accessed on 1 November, 2023)) was utilized to examine the trends, patterns of public interest and search interest landscapes in nephrolithiasis [37]. We analyzed the search trends to identify stationarity of the trends and to predict the future trends.

### 2.1. Examining Search Trends with Google

Google Trends is a free, easily accessible and popular tool for analyzing search engine queries. It offers valuable insights into public health topics due to its real-time search volume data, as evidenced by its use in tracking influenza outbreaks and monitoring e-cigarette interest [38,39]. This allowed us to track fluctuations in interest for “kidney stone” over nearly two decades (January 2004–November 2023), the longest timeframe available through Google Trends.

This study applied the term “kidney stone”, subtype “disease”, and region “worldwide” into the Google Trends tool. We selected the “All categories” option to avoid limiting the search to any particular category. The “Web Search” option was then selected in order to retrieve results from January 2004 to November 2023. After assigning the search term and criteria, information regarding overall monthly relative search volume (RSV) and geographical distribution of search interest, along with its RSV, was displayed. 

Google Trends reports the popularity of search queries over time on a monthly basis by estimating the RSV of searches made by Google users. The RSV is presented on a scale from 0 to 100, where 100 represents the peak popularity of a term within a selected time frame and geographic region. For instance, an index of 50 means that the search activity for the term is relatively half of its peak level [37]. 

### 2.2. Stationarity Testing

A time series is data collected over time, e.g., annual global temperature or stock prices. It can be stationary or non-stationary. A non-stationary time series means the statistical properties (such as mean, variance, and autocorrelation) are time-dependent, while a stationary time series means these statistical properties are constant over time. The Augmented Dickey–Fuller (ADF) test is a statistical tool designed to assess whether a time series data is stationary. The null hypothesis of the ADF test is that there is a unit root in an autoregressive time series model. A unit root refers to a specific characteristic that affects the data pattern over time, and a time series with a unit root is considered non-stationary. The alternative hypothesis is that the series is stationary. The ADF test is a modification of the Dickey–Fuller test, where the ADF eliminates autocorrelation from the series and then tests in a similar way to the Dickey–Fuller test method. A *p*-value less than 0.05 indicates significant stationarity. This significant statistical finding will determine the reliability of the time series forecasting analysis. Thus, we performed the ADF test among the monthly search indices of “kidney stone” and their relative change over time to determine whether the search interest in “kidney stone” from 2004 to 2023 was stationary.

### 2.3. Forecasting Search Trends

After determining stationarity of the time series, we employed the autoregressive integrated moving average (ARIMA) model to predict future trends in “kidney stone” search queries. The ARIMA model combines the autoregressive (AR) model, the integrated (I) model, and the moving average (MA) model to better adapt and predict time series data that shows non-stationarity or trends. In addition to forecasting the trends, utilizing ARIMA allowed us to anticipate potential fluctuations in public interest related to kidney stones over time.

### 2.4. Statistical Analysis

Our data analysis was conducted using Python’s pandas library and visualizations were created with Seaborn, enhancing the interpretability of our findings. This study used Google Trends data that were compliant with Google’s privacy policy (www.google.com/privacypolicy.html (accessed on 1 November 2023)). The data did not contain any personal identifiers, such as names, IP addresses, or exact location data to track individual users, thus ensuring confidentiality of this online search [37]. We used ChatGPT-4 to generate accurate Python code snippets under our direct supervision. ChatGPT-4 served to expedite our data processing, while the original idea, findings, data interpretation and conclusion originated from our team.

## 3. Results

### 3.1. Global Google Search Interests Trends

**Figure 1:**
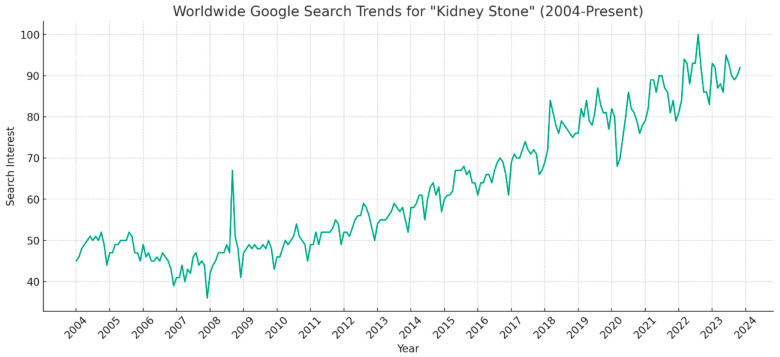
Google Search Trends for “Kidney Stone” from 2004 to 2023.

Worldwide Google search interest for “kidney stone” showed an overall upward trend since its first recording in 2004, with an RSV score of 48. Within the study period of January 2004 to November 2023, the mean search interest RSV score was 64.25 ± 15.3 (Figure 1). Notable fluctuations in interest were present within this overall positive pattern. Initially, there was a gradual decline in search interest, reaching its lowest point of 36 in December 2007. This was followed by an initial sharp spike in May 2008, with a score reaching 67. Following this temporary surge, the interest returned to its baseline level of around 48. Thereafter, a sustained upward trend began, progressively increasing from a low of 41 in December 2008 to a peak of 92 in November 2023. The highest search interest peak was in August 2022 (RSV 100). 

### 3.2. Stationarity of Trends

The search interest for “kidney stone” exhibited initial fluctuations of around 10 points (mean 48 ± 10.2) from 2004 to 2018. Subsequently, the trend became more dynamic with variations of up to 20 points (mean 70 ± 20.4). As displayed, the highest annual interest peak tended to occur in the latter half of each year, from August to October, during 2004–2016. From 2017 onwards, the annual peak shifted earlier, to between March and June. Each peak was typically followed by a gradual decline, reaching its lowest point by the respective year’s end (Figure 1).

However, the Augmented Dickey–Fuller (ADF) analysis revealed a *p*-value of 0.023, suggesting stationarity in the series. This implies that, despite the observed fluctuations, the overall public interest in “kidney stone” remained statistically constant, with no significant trend of increase or decrease. 

### 3.3. Predicted Future Trends in “Kidney Stone”

**Figure 2:**
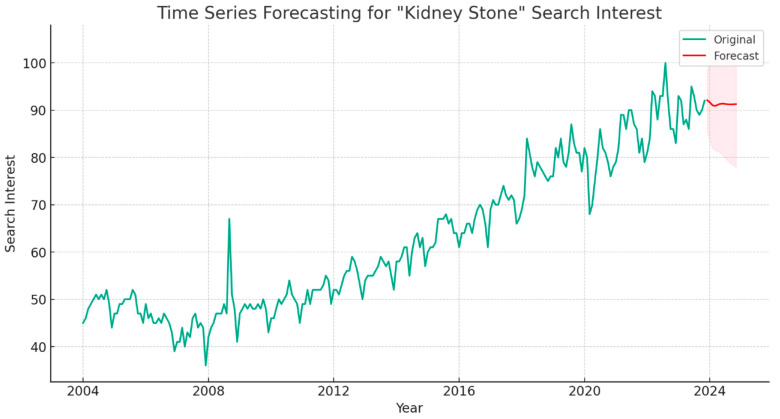
Time Series Forecasting for “Kidney Stone” Trend in 2024.

Times series forecasting analysis predicted a sustained high level of global interest in “kidney stone”, with values consistently exceeding 90 throughout 2024 (Figure 2). 

### 3.4. Geographic Distribution of Interest

**Figure 3:**
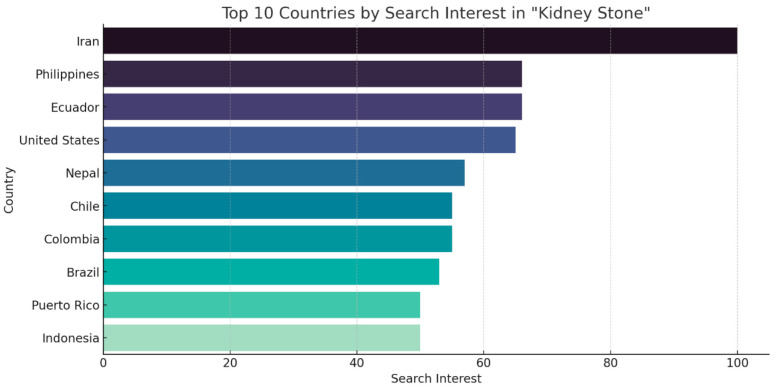
Google Search Trends by Region for “Kidney Stone” from 2004 to 2023.

Among the top 10 countries with the highest “kidney stone” search interest during 2004–2023, Iran emerged as the frontrunner, with a maximum RSV score of 100 (Figure 3). The remaining countries displayed more moderate search interest in comparison. The Philippines and Ecuador share similar levels at around 65, followed closely by the United States at 64 and Nepal at 56. Chile and Colombia exhibit comparable scores at 55, while Brazil was slightly lower at 52. Puerto Rico and Indonesia were at the bottom of this list with scores of 50 each (Table 1).

We assessed the number of scholarly publications on the “kidney stone” topic from the top 10 countries with the highest respective search interest as shown in Table 1. The United States had the highest number of publications with 1746 articles, with Iran following with 438 articles, and then Brazil with 215 articles. The Philippines, Ecuador, Nepal, Chile, Colombia, Puerto Rico and Indonesia had publications ranging from 3 to 66 articles (Figure 3). The predominant climate in many of the aforementioned countries is tropical, marked by warm and humid conditions throughout the year but particularly during the June to August period. Iran, known for its arid climate, had the highest search interest for kidney stones. The seasonal breakdown listed in Table 1 provides an overview of the expected weather conditions. From June to August, the majority of these countries, such as Iran, the Philippines, the United States, Nepal, Puerto Rico and Indonesia, experience summer and high temperatures. Others have dry or winter seasons during this timeframe, such as Ecuador, Chile, Colombia and Brazil. From September to November, December to February, and March to May, the majority of these countries appear to have cooler and rainy weather, compared to the overall weather from June to August [40]. This seasonal variation of weather can be correlated with search interest. 

## 4. Discussion

This study utilized the online Google Trends tool via the Google search term “kidney stone” from 2004 to 2023 as a surrogate marker of public interest in nephrolithiasis. Our results showed a significantly increasing trend in interest about “kidney stone”, particularly during the last decade. The peak search interest was from 2022 to 2023. Based on the findings of a time series forecast analysis, it is expected that the search volume for the year 2024 will be consistent with the previous year.

The Google Trends’ measurement of search interest is tailored to yield insights that extend beyond mere volume of searches. It employs an indexing system that benchmarks the search interest for a term against the term’s highest point of popularity within a specific region and timeframe. Thus, a score of 100 signifies the all-time zenith of search interest for the term, while a score of 50 indicates that the term’s popularity is at half of its peak. This indexing is critical as it presents each data point as a ratio of the subject’s total searches in relation to all Google searches carried out in the same location and time period. The effect of this is twofold: (1) it allows for equitable comparison of interest levels in diverse geographical areas, which may have different total search volumes due to population variance or differing degrees of internet accessibility, and (2) it ensures that the search interest reflects the search frequency about a topic relative to all other topics being queried, when the comparisons are added at the same time [42,43,44,45]. As a result, the size of a country or its population impacts the absolute number of searches but not the search interest index as reported by Google Trends. This relative measure of search interest is crucial for understanding the actual level of awareness or concern in a region, as it removes the bias of absolute numbers that are naturally higher in countries with larger populations and more internet users. For instance, while the United States may have the greatest number of publications and a vast population potentially contributing to search volumes, it is the proportionate interest—how many searches are made for “kidney stone” relative to all searches—that Google Trends captures and reflects in its index. This proportional representation explains why a country like Iran, possibly with fewer academic publications on kidney stones or a smaller populace, can exhibit a high search interest in the condition. The indexed value thus offers a nuanced perspective on public interest, allowing for an analysis that appreciates the intensity of search activity relative to the size of the country and its internet usage patterns. While the rising search volume for “kidney stone” could be linked to a genuine increase in internet access, other possible contributing factors are proposed as follows.

### 4.1. Increase of Kidney Stone Prevalence

The increasing search volume could be correlated with the rising prevalence and incidence of kidney stones globally over the past decade. Multifactorial pathophysiologic factors [20,21,46] may have affected the prevalence and incidence of kidney stones [5,6,15,16]. These include advances and improved availability of medical technology and imaging modalities for earlier detection and diagnosis, climate change, and a shifting individual and societal dietary, metabolic, and lithogenic environment. Furthermore, improved education and increasing availability of the internet might motivate individuals to seek knowledge about this disease, leading to a significant increase in search volumes. Finally, social media and various factors such as public health system campaigns, influencers, drug advertisements, and marketing can all significantly impact the search volume.

### 4.2. Role of Work Environment

Environmental and occupational factors impact kidney stones and translate to public awareness. Certain occupations exposed to high temperatures have been shown to exacerbate stone risk, such as those in the steel industry. Workers exposed to high temperatures faced a nine-fold increased risk of urolithiasis, with observed metabolic changes including hypocitraturia and low urine volumes [47].

### 4.3. Role of Climate Change 

Climate change has been identified as a significant environmental factor influencing the prevalence and incidence of kidney stones [25,26]. The rate of global temperature rise since 1970 has been the fastest in any 50-year interval over the past 2000 years. The global surface temperature from 2011–2020 was 1.09 °C warmer than in 1850–1900 [48]. A review by Spiardi et al. states that kidney stones appear to be more prevalent in urban areas and also areas that are hotter and more humid, with factors such as humidity and sweat loss influencing stone formation. This risk is further amplified after periods of high heat, and men seem to be more susceptible compared to women [25]. The relationships between climate, kidney stone risk, and public interest in kidney stones is observed among the top 10 countries with the highest search interest. Iran, characterized by an arid climate, surpassed the others, with the highest search interest for kidney stones. The majority of the other countries have tropical climates, where the weather is warm and humid throughout the year, especially from June to August (6 out of 10 countries). This corresponds to the observation that the peak of interest in each year was more likely to occur in the second half of the year, mostly in August to October. After reaching the annual peak interest, the RSV tended to decrease gradually until the end of the year. This trend correlates with the time when cooler weather conditions occur in the majority of the top 10 countries (Table 1).

### 4.4. Dietary and Lifestyle Changes

Global awareness of the multifaceted and pervasive effects of dietary changes on the world population is another exacerbating factor to increasing levels of kidney stones. Dietary changes, especially increased consumption of fast foods and preparations containing high fructose corn syrup, have been identified as a major factor contributing to the prevalence of kidney stones [23]. Data from the US Department of Agriculture spanning from 1909 to 1997 shows an increase in the proportion of total energy consumption of dietary carbohydrates, such as corn syrup, from 48% to 54% [24]. Substantial shifts in dietary habits have also been observed across the world, especially during the COVID-19 pandemic [49,50]. A study by Tavasoli et al. examined the impact of the COVID-19 pandemic on urinary stone profiles in nephrolithiasis patients in Iran. Comparing pre- and post- pandemic, changes in 24-hour urine metabolites demonstrated higher magnesium and lower urea, sodium, and potassium levels. These findings suggest potential shifts in dietary habits during the pandemic, possibly influenced by economic factors or restricted access [51]. Moreover, osteoporosis was found to be a significant risk factor for nephrolithiasis [27]. Osteoporosis and kidney stones are both diseases associated with unhealthy lifestyles [52]; one of the common risks is having negative calcium balance [53], which increased the risks of both diseases. Inadequate dietary calcium intake reduces formation of calcium and oxalate salts in the intestinal lumen, resulting in increasing urinary oxalate excretion and calcium oxalate stones [54].

### 4.5. Imaging Advancements and Diagnosis Impact on Kidney Stone Trends

Regarding the increasing widespread availability of accurate diagnostic imaging [18], studies have demonstrated an increased utilization of computed tomography (CT) scans [55,56] and ultrasounds [57]. CT scans exhibit high sensitivity and specificity in detecting stones, even very small ones [58]. Moreover, the routine use of both stationary and bedside ultrasound devices, which are becoming increasingly accessible and integrated as part of routine clinical care, can also identify both asymptomatic and symptomatic nephrolithiasis [59,60]. Research on the prevalence, incidence, and recurrence of kidney stones has often yielded inconsistent results, however. This is partly due to a lack of a standardized stone classification system [18]. Thus, one possible explanation for the diverse variation in search interest across regions could be the differences in imaging modalities’ availability and the criteria utilized for diagnosing kidney stones.

### 4.6. Social Media and Celebrity Influence

Social media has had ubiquitous effects on medicine. There have been several notable cases of kidney stones in popular culture that likely contributed to the public’s interest and awareness of the condition. For instance, in January 2006 the renowned “Star Trek” actor William Shatner made headlines when he sold his kidney stone for USD 25,000, setting a Guinness World Record for the most expensive kidney stone ever sold [61]. This unusual news story garnered significant media attention, potentially sparking public curiosity and subsequent information searches about kidney stones. In another high-profile incident in 2009, Gene Simmons, the iconic bassist of the band KISS, raised USD 15,000 for charity by auctioning off his kidney stone on eBay. He captured public interest again in 2023 when he revealed in a backstage interview that he was managing a kidney stone during his performance at the KISS farewell show [62]. Such events involving celebrities can influence public awareness and lead to peaks in search trends, as was observed in 2009, which coincided with Simmons’ initial news (Figure 1). Furthermore, the advent of social media platforms has drastically increased the avenues through which awareness about medical conditions like kidney stones can be spread. Platforms such as Facebook have facilitated the creation of online communities where individuals can share their experiences. For example, groups dedicated to discussing kidney stones provide a space for thousands of members to exchange advice, personal stories, and support. The existence of such a group with over 30,000 members dedicated to kidney stones underscores the significant role that social media plays in raising public awareness [63]. The cumulative effect of these cultural touchstones, coupled with the enhanced connectivity provided by social media, likely influences the patterns observed in Google search trends. The long-term data from 2004 to 2023, which are shown to be statistically stationary, allow for robust analysis and forecasting. The stationarity of the dataset suggests that the level of public interest in kidney stones has remained relatively consistent over time, enabling predictive models to forecast future trends with a degree of confidence. These predictive insights can be particularly useful for healthcare professionals, policymakers, and educators as they plan interventions and resources to address the information needs related to kidney stones.

### 4.7. Evolution of Kidney Stone Treatments

The field of kidney stone interventions has had remarkable evolutions, transitioning from conventional open surgeries in the early 1990s to minimally invasive techniques such as percutaneous nephrolithotomy and ureteroscopy currently. The popular advancements in this area include the development of laser technologies like the holmium: yttrium–aluminum–garnet (Ho:YAG) and the thulium fiber laser [36]. These ongoing innovations in surgical treatment could also contribute to significant public interest. For example, Ho:YAG laser lithotripsy is now viewed as one of the most secure and efficient methods for treatment of urinary stones [64,65,66]. The development of the Ho:YAG laser revolutionized endoscopic treatment of stones over 15 years ago [36], which may be ascribed to the initial peak global search interest in 2008 (Figure 1). The Ho:YAG laser remains the gold standard for intervention, recommended by the European Association of Urology Guidelines [67]. The incorporation of artificial intelligence (AI) has further enhanced stone treatment by aiding in diagnosis, treatment planning, and prediction of kidney stone outcomes [36,68]. These advancements may have raised public awareness.

### 4.8. Potential Implications

The rising prevalence of global awareness of kidney stones over the past 20 years underscores the need for AI-powered search engines. By incorporating short surveys to understand user intent, e.g., initial symptoms, diagnosis, or treatment, and leveraging generative AI, search results can be personalized. This could include tailored treatment options, lifestyle modifications, or even emergency alerts based on a user’s situation. While challenges including data privacy and AI bias exist, this user-centric approach with AI has the potential to empower individuals, leading to better health literacy, earlier diagnoses, and improved kidney stone management. The analysis of Google search trends regarding “kidney stone” can be instrumental for healthcare professionals and policymakers in identifying periods of heightened concern and targeting health interventions to the right time, e.g., focusing on education, prevention, and management strategies. The integration of Google Trends analysis into healthcare planning exemplifies the intersection of technology and medicine, offering a proactive approach to public health monitoring and resource allocation. This study brings novel contributions to the analysis of kidney stone trends. This could be a future strategy for preventive medicine, as we employed Google Trends data in an innovative manner to examine public interest in kidney stones. We also utilized time series forecasting analysis to predict future trends in global interest, aiding healthcare professionals and policymakers in addressing public health concerns. Moreover, the study explored various elements contributing to the observed trends, thereby enriching our understanding of the dynamics driving public interest in nephrolithiasis.

### 4.9. Limitations

Our study has some limitations. First, due to the limitation of data from Google Trends, we could not evaluate in-depth factors or analyze pre-specified subgroups that may have impacted the data’s robustness, such as age, gender, socioeconomic status, and potential internet access barriers. Second, our search was limited to keywords using only the English version, which may result in missing data from other languages. Third, this study only evaluated the search trends from one popular search engine, and thus it may not reflect the information-seeking behavior on other platforms or channels. Fourth, technology has transformed our lifestyle dramatically in the last 20 years. We did not evaluate the traditional or alternative methods that individuals use to search for information, which may vary across regions. Therefore, the results of this study may have limited generalizability. Fifth, it is clear that search interest is not a comprehensive measure of public engagement or the quality of the information obtained. We did not evaluate the knowledge acquisition of the general population for the causes, symptoms, and treatments of kidney stone disease. This may be an important area for future research when designing effective media, modalities, and tools for patient education. Sixth, the digital landscape of health information-seeking behavior can be influenced by external factors, such as changes in the algorithms of search engines. Lastly, the time series forecasting analysis is not a validated method, and thus its projected forecasts can have varying levels of accuracy.

## 5. Conclusions

Kidney stones are causing a significantly increased healthcare burden worldwide. The global search interest in “kidney stone” has been stable and increasing for the past two decades across different regions worldwide, with projections for continued interest in the future. This public interest may be attributed to multifactorial elements, such as increased global warming, lifestyle and dietary changes, advancements in healthcare, social media, and improving education and internet availability. This study’s analysis serves as a useful resource for shaping future healthcare policies and research directions to address kidney stone-related health challenges and meet projected public interest. The increasing search interest highlights the need for healthcare leaders worldwide to ensure accurate, updated, and peer-reviewed healthcare information on the internet is available to the public and future artificial intelligence models. Furthermore, communication and information technology experts are needed to ensure that navigating to the certified information is easy to do and that the available information on the internet is reliable. 

## Figures and Tables

**Table 1:** Comparison of search interest, number of publications, overall climate and seasons, and seasonal characteristics per quarter of the year among the top 10 countries with the highest kidney stone search interest [40,41].

Countries/ Continent	Search Interest	Publications during 2004–2023	Overall Climate	Seasons with Hot and Humid Weather			
				December–February	March–May	June–August	September–November
Iran/ Asia	100	438	Arid to sub-tropical	−	+	++	−
Philippines/ Asia	65 *	14	Tropical and maritime	−	+	++	++
Ecuador/ South America	65 *	2	Tropical	+	+	−	+
United States/ North America	63 *	1746	Varied	−	+	++	−
Nepal/ Asia	57 *	66	Tropical to alpine	−	+	++	−
Chile/South America	55 *	31	Varied	++	−	−	+
Colombia/ South America	55 *	17	Tropical Marine	−	+	−	+
Brazil/ South America	52 *	215	Tropical	++	−	−	+
Puerto Rico/ North America	50 *	3	Tropical	−	++	++	−
Indonesia/ Asia	50 *	49	Tropical	−	+	++	−

* The information on this table consists of general descriptions and actual weather conditions may vary based on specific regions within each country and year-to-year variations. The number of publications from each country was collected through a PubMed search utilizing the keywords “[country name] + kidney + stone” for any publications spanning the period 2004–2023. “++” refers to both hot and humid weather, such as in summer and monsoon. “+” refers to either hot or humid weather, or mildly hot and humid weather, such as spring. “−” refers to both dry and cool/cold weather, such as in winter or fall.

## Data Availability

The original contributions presented in the study are included in the article, further inquiries can be directed to the corresponding author.
